# Penfluridol overcomes paclitaxel resistance in metastatic breast cancer

**DOI:** 10.1038/s41598-019-41632-0

**Published:** 2019-03-25

**Authors:** Nehal Gupta, Parul Gupta, Sanjay K. Srivastava

**Affiliations:** 1 Department of Biomedical Sciences, Texas Tech University Health Sciences Center, Amarillo, TX 79106 USA; 2 Department of Immunotherapeutics and Biotechnology, and Center for Tumor Immunology and Targeted Cancer Therapy, Texas Tech University Health Sciences Center, Abilene, Texas, 79601 USA

## Abstract

Paclitaxel is a first line chemotherapeutic agent for the patients with metastatic breast cancer. But inherited or acquired resistance to paclitaxel leads to poor response rates in a majority of these patients. To identify mechanisms of paclitaxel resistance, we developed paclitaxel resistant breast cancer cell lines, MCF-7 and 4T1 by continuous exposure to paclitaxel for several months. Western blot analysis showed increased expression of HER2 and β-catenin pathway in resistant cell lines as compared to parent cells. Hence, we hypothesized that HER2/β-catenin mediates paclitaxel resistance in breast cancer and suppression of HER2/β-catenin signaling could overcome paclitaxel resistance. Our data showed that penfluridol (PFL) treatment significantly reduced the survival of paclitaxel-resistant cells. Western blot analysis revealed that PFL treatment suppressed HER2, as well as, β-catenin pathway. *In vivo* data confirmed that PFL significantly potentiated tumor growth suppressive effects of paclitaxel in an orthotropic breast cancer model. In addition, tumors from paclitaxel and PFL-treated mice showed reduced HER2 and β-catenin expression, along with increased apoptosis. Taken together our results demonstrate a novel role of HER2/β-catenin in paclitaxel resistance and open up new avenues for application of PFL as a therapeutic option for overcoming paclitaxel resistance.

## Introduction

Breast cancer remains the second leading cause of cancer related mortality in women, despite the advances in treatment strategies. In 2017, according to American Cancer Society, about 40,610 women were expected to die of breast cancer^1^. Patients with metastatic breast cancer have only 5% survival rate, indicating metastasis as the major contribution to breast cancer mortality rate^2,3^. Taxanes, including paclitaxel are approved and clinically used chemotherapeutic agents for the treatment of early and advanced metastatic breast cancer^4–6^. However, response rate of taxanes for metastatic breast cancer ranges from 30–70%^7^. Literature suggests, over 90% of patients of unresponsive patients have inherited or acquired resistance to the therapy^8^. Only few studies suggest a role of PI3K/Akt, FOXK2 and transgelin in paclitaxel resistance^9–13^. Due to large gaps in understanding of paclitaxel resistance mechanisms, therapeutic benefits have been limited. Therefore, more research into molecular mechanisms underlying drug resistance is essential for the development of improved therapies.

Human epidermal growth receptor 2 (HER2) amplification is observed in about 30% of breast cancer patients and is correlated with poor disease prognosis^14,15^. There is a considerable debate about HER2 overexpression and taxane sensitivity in breast cancer cells. Several clinical studies have suggested the role of HER2 amplification in inducing chemotherapeutic resistance^16–18^. In stark contrast, other studies have shown better response rate to taxanes in patients with HER2 positive tumors^19–21^. Therefore, there is a considerable need to validate the function of HER2 and to elucidate the mechanisms that underplay downstream of HER2 in altering taxane sensitivity in breast cancer.

A clinical study has shown correlation between HER2 and β-catenin leading to poor prognosis in breast cancer patients^22,23^. In addition, β-catenin plays role in cell response to paclitaxel treatment and also in tamoxifen resistance in breast cancer^24,25^. Therefore, we hypothesized interplay of HER2 and β-catenin in breast cancer resistance to paclitaxel.

β-catenin is a multifunction protein, which has been shown to perform dual functions; playing a crucial role in maintaining physiological homeostasis and also functioning as an oncogene^26,27^. The constitutive activation of β-catenin signaling in several malignancies^26^ including breast cancer^28^, makes it a potential target for therapy^29^. Dysregulation of β-catenin signaling in breast cancer results in cell proliferation, tumor initiation, progression and metastasis^30^. In addition, Wnt/β-catenin signaling has also been associated with the development and differentiation of cancer stem cells^31,32^. To this end, no study has clearly demonstrated the involvement of Wnt/β-catenin signaling towards taxane resistance in breast cancer.

In the present study, we have developed paclitaxel resistant cells by continuous exposure to paclitaxel for several months. The cells were analyzed for molecular changes as compared to parent cell lines. The resistant cell lines showed increased expression of HER2 and β-catenin as compared to parent cells. Herein, we demonstrate a direct role of HER2 in acquired resistance to paclitaxel, via β-catenin signaling. The downregulation of HER2/β-catenin signaling resulted in increased sensitivity of breast cancer cells to paclitaxel. Furthermore, based on our previous observations we evaluated efficacy of penfluridol (PFL), a neuroleptic agent, in paclitaxel resistant cells^33–36^. Our data showed that combination of PFL with paclitaxel suppresses paclitaxel resistant breast tumor growth and inhibits HER2/β-catenin. Overall, this study provides a crucial insight into role of HER2 in paclitaxel resistance mediated by β-catenin, and which can be reversed by a neuroleptic agent PFL.

## Results

### Paclitaxel-resistant cells exhibit significantly low sensitivity towards paclitaxel as compared to parent cells

**Figure 1:**
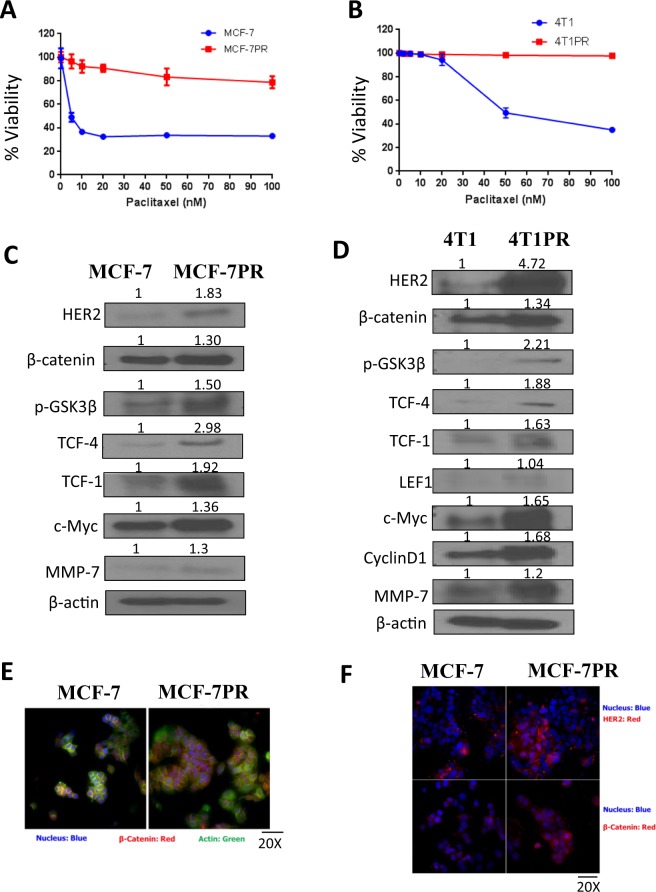
Development of paclitaxel resistance in MCF-7 and 4T1 cells show induced expression of HER2 and β-catenin. MCF-7 and 4T1 cells were treated with gradually increasing concentrations of paclitaxel for several months. Cytotoxicity of (**A**) MCF-7 vs MCF-7PR and (**B**) 4T1 vs 4T1PR cells induced by paclitaxel after 72 hour treatment to estimate the percent cell survival. Experiments were repeated three times with 8 replicates in each experiment; Data shown as mean ± SD. (**C**) Relative expression of proteins in MCF-7 and MCF-7PR cells. (**D**) Relative expression of proteins in 4T1 and 4T1PR cells. Experiments were repeated at least three times. (**E**) Immunofluorescence images showing morphological differences between MCF-7 and MCF-PR cells. (**F**) Comparison of HER2 and β-catenin expression in MCF-7 and MCF-7PR cells by Immunofluorescence. Red fluorescence, HER2 or β-catenin; Green, actin; Blue, DAPI.

The MCF-7, and MCF-7PR were treated with increasing concentrations of paclitaxel and their viability was evaluated via Sulforhodamine B (SRB) assay. The IC_50_ for paclitaxel in MCF-7 cells was found to be 5 nM (Fig. 1A). Following this, MCF-7 cells were continuously exposed with low-dose of paclitaxel for several months by a stepwise increase in the concentration (2.5 to 300 nM). The IC_50_ was not achieved in MCF7-PR cells even at 100 nM paclitaxel. At 100 nM paclitaxel, viability of MCF-7 cells was about 30%, whereas for MCF-7 PR cells it was 80% relative to untreated control wells (Fig. 1A). This observation was also confirmed in a highly aggressive cell line 4T1 and its variant 4T1-PR. The IC50 in parent 4T1 cells was 50 nM, whereas IC50 was not achieved in 4T1-PR cells (Fig. 1B). Only about 35% 4T1 cells survived after treatment with 100 nM paclitaxel. On the other hand survival in 4T1-PR cells after 100 nM paclitaxel was 100%, relative to untreated control wells. This data confirmed that MCF-7PR and 4T1PR breast cancer cells had acquired resistance to paclitaxel.

### HER2 and β-catenin signaling is activated in paclitaxel resistant cells

To identify possible mechanism of resistance, basal expression of several proteins was evaluated in paclitaxel sensitive and resistant cells by western blotting. As compared to the parent cell, the MCF-7PR and 4T1PR cells showed increased expression of HER2 and β-catenin along with higher expression of its downstream molecules, such as, TCF-4, TCF-1, p-GSK3β, Cyclin D1 and c-Myc (Fig. 1C,D). These observations suggested a role of HER2 and β-catenin signaling in the development of resistance to paclitaxel.

### MCF-7PR cells show morphological differences as compared to parent cells with increased expression of HER2 and β-catenin

The MCF-7 and MCF-7PR cells were analyzed for the differences in morphology and protein expression of HER2 and β-catenin using fluorescent microscopy. The morphology of cells was analyzed using actin and DAPI staining. Our results showed that the resistant cells were relatively enlarged and more importantly β-catenin was localized in the nucleus of the cells as compared to parent cells, where β-catenin co-localized with actin in the cell membrane (Fig. 1E). In line with above observations, imaging also showed an increased expression of HER2 in MCF-7PR cells, as compared to MCF-7 cells (Fig. 1F). This data confirmed variations in expression of HER2 and β-catenin as compared to the parent cells.

### Role of HER2 in paclitaxel resistance

**Figure 2:**
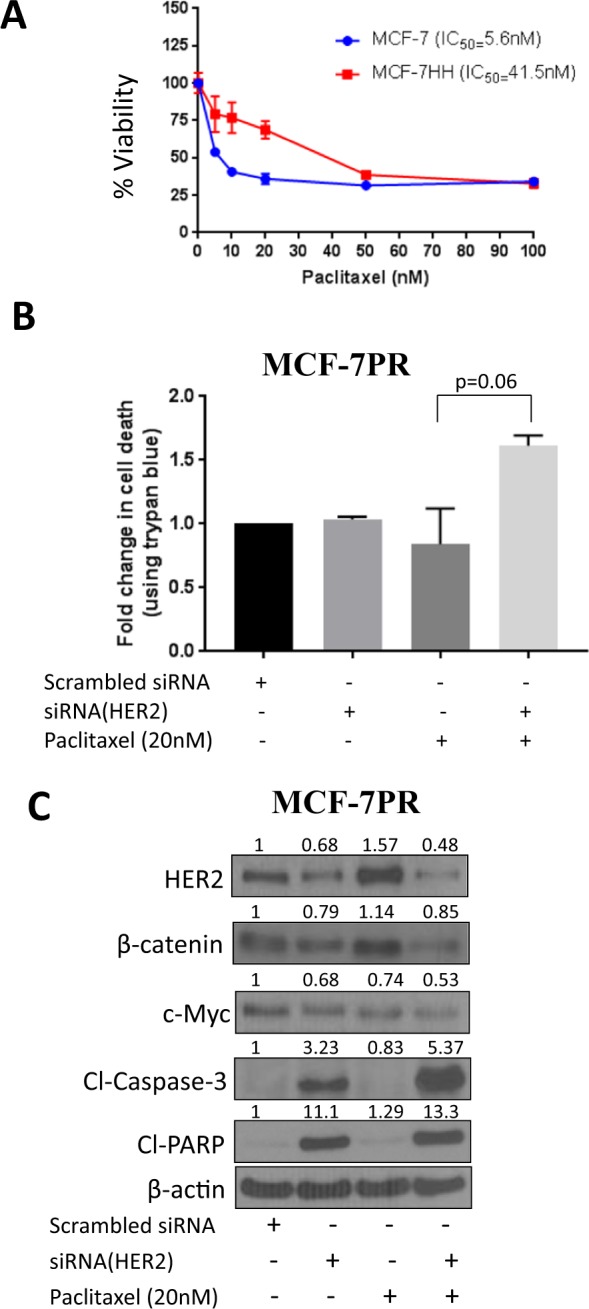
HER2 plays a crucial role in imparting paclitaxel resistance. (**A**) Comparison of paclitaxel cytotoxicity in MCF-7 and MCF-7HH cells via SRB assay after 72 hours paclitaxel treatment; Data shown as mean ± SD. (**B**) Cell death analysis using trypan blue and (**C**) Western blot analysis in MCF-7PR cells treated with paclitaxel (20 nM) for 72 hours after transfection with either HER2 siRNA or scrambled siRNA. Statistical differences were calculated by Student’s *t* test.

To further establish the role of HER2 in paclitaxel resistance, we evaluated paclitaxel cytotoxicity in MCF-7 HER2 overexpressing cells (MCF-7HH) cells using SRB assay. MCF-7HH cells are parent MCF-7 cells with stable HER2 overexpression. Our results showed about 8 fold higher IC_50_ in MCF-7HH cells relative to parent MCF-7 cells (Fig. 2A). To further confirm the role of HER2 in resistance, we genetically knocked down HER2 in MCF-7PR cells using HER2 siRNA. We observed that silencing of HER2 resulted in increased sensitivity of MCF-7PR towards paclitaxel as indicated by increased cell death in cells transfected with HER2 siRNA and treated with paclitaxel (Fig. 2B). Our results also showed that silencing HER2 also suppressed the levels of β-catenin and c-Myc, and that the expression of these proteins was reduced further by paclitaxel treatment (Fig. 2C). These observations clearly suggest a role of HER2/β-catenin in paclitaxel resistance.

### Cross talk between HER2 and β-catenin

**Figure 3:**
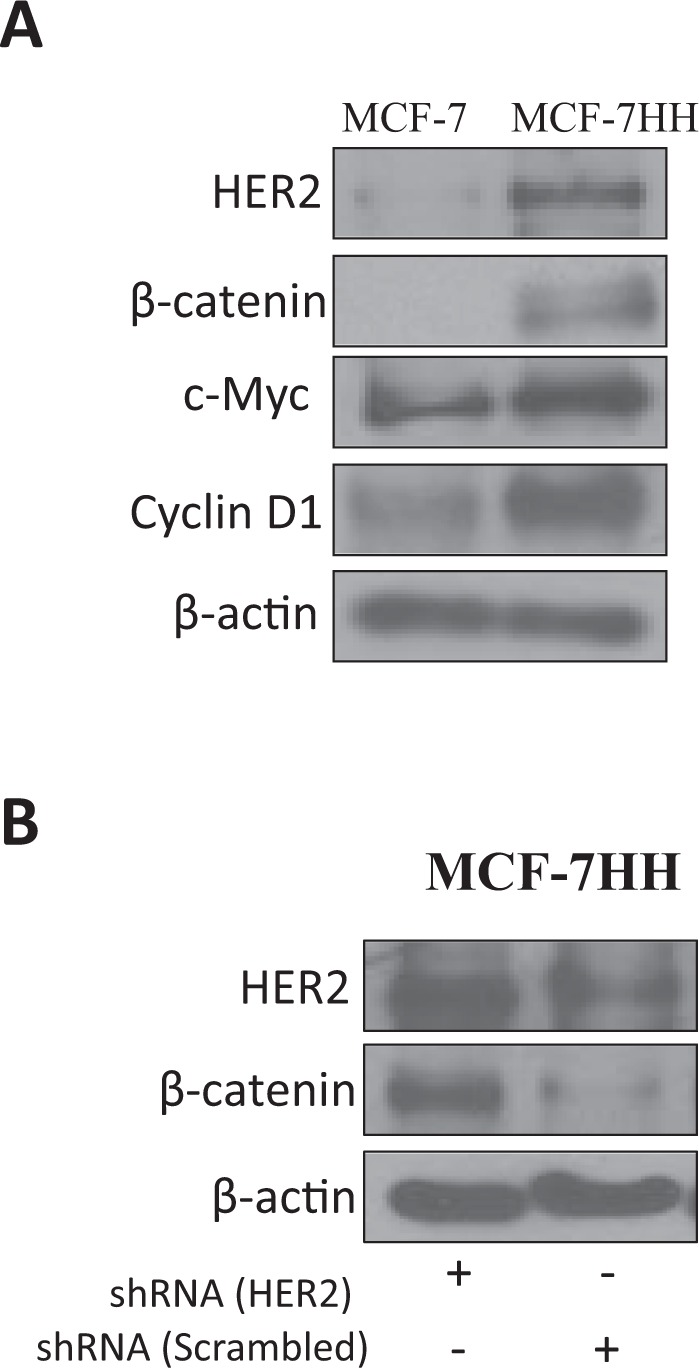
Cross talk between β-catenin and HER2. (**A**) Basal level of markers in MCF-7 and MCF-7HH cells. (**B**) Levels of HER2 and β-catenin in MCF-7HH cells transfected with either HER2 shRNA or scrambled shRNA for 24 hours as determined by Western blotting.

Our results had showed increased expression of HER2 and β-catenin in both the drug resistant cell lines, suggesting possibility of cross-talk between HER2 and β-catenin. To explore this further, the expression of β-catenin pathway was compared in parental MCF7 and MCF7-HH cells. Strikingly, the cells expressing high level of HER2 (MCF-7HH) also showed higher expression of β-catenin and its downstream effector molecules, such as, c-Myc and cyclin D1 (Fig. 3A). In addition, when HER2 was silenced using shRNA in MCF7-HH cells, a corresponding reduction in the expression of β-catenin was seen (Fig. 3B). Overall our findings so far, indicate a cross-talk between HER2 and β-catenin pathway that contributes to paclitaxel resistance.

### PFL reduces the survival of resistant cells by downregulating the expression of HER2 and β-catenin

**Figure 4:**
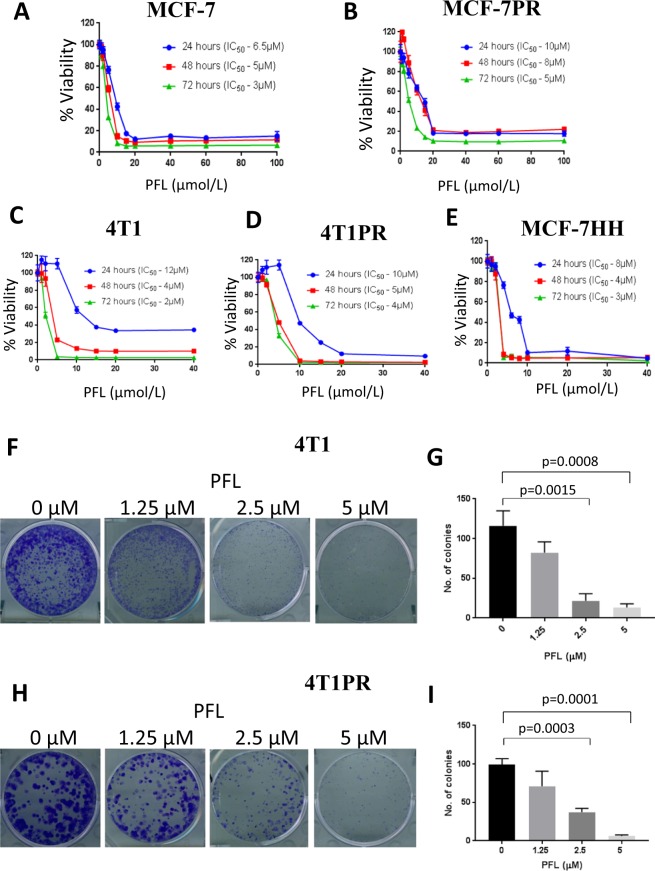
PFL suppresses cell survival of paclitaxel sensitive and resistant cells. (**A**) MCF-7, (**B**) MCF-7PR, (**C**) 4T1, (**D**) 4T1PR, and (**E**) MCF-7HH cells were treated with different concentrations of PFL at 24, 48 and 72 hours. Cell survival was measured by SRB assay to estimate IC_50_ values. The experiments were repeated three times with 8 replicates in each experiment. Colony formation assay was performed by seeding 500–600 cells/well. Cells were fixed and stained using crystal violet (0.5%) after 9 days. Number of colonies formed in control and PFL treated wells were quantitated using Image J software. Representative Images of colonies and their quantification in (F-G) 4T1 cells and (H-I) 4T1PR cells. Data shown as mean ± SD; (n = 3). All the statistical comparisons were made by Student’s *t* test for unpaired samples.

So far, we identified that HER2 and β-catenin signaling gets activated in paclitaxel resistant cells. Hence, we hypothesized that HER2/β-catenin signaling mediates paclitaxel resistance in breast cancer and inhibiting this oncogenic signaling could overcome resistance to paclitaxel. In a previous study, we demonstrated that PFL, an anti-psychotic drug suppresses the growth of triple negative metastatic breast cancer cells^35^. Therefore, we evaluated the growth suppressive effects of PFL in paclitaxel sensitive and resistant cells. First, we performed the cytotoxicity assay in MCF-7, MCF-PR, 4T1 and 4T1PR cells. The cells were treated with varying concentrations of PFL for 24, 48 and 72 hours. Our results showed that increasing concentrations of PFL significantly suppressed the growth of not only paclitaxel sensitive cells but also paclitaxel resistant cells in a concentration and time-dependent manner. The IC_50_ values of PFL ranged 2–3 μM in paclitaxel sensitive cell lines (MCF-7, 4T1) and 4–5 µM in paclitaxel resistant cell lines (MCF-7PR, 4T1PR) after 72 hours of treatment (Fig. 4A–D). In addition, we also evaluated the cytotoxic effects of PFL in MCF-7HH cells and the IC_50_ was about 3 μM at 72 hour time point (Fig. 4E). These results suggest PFL is almost equally toxic to paclitaxel sensitive and resistant cells. We also conducted colony formation assay to investigate whether PFL could inhibit the proliferation capacity of breast cancer cells. Results from this assay clearly showed that PFL treatment significantly (p = 0.0001–0.0015) decreased the size and number of colonies in a concentration-dependent manner (Fig. 4F–I).

**Figure 5:**
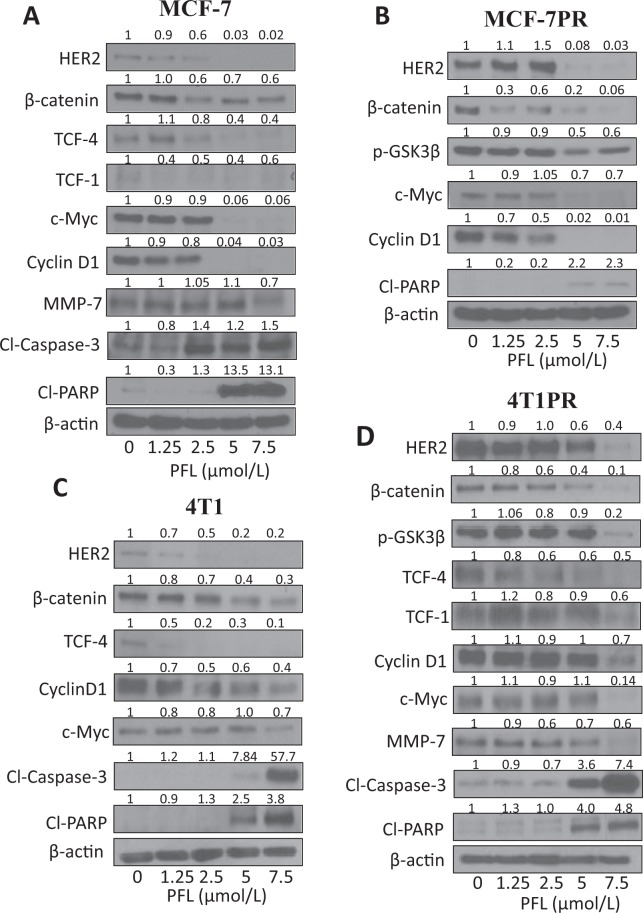
Suppression of HER2/β-catenin and associated signaling by PFL treatment. (**A**) MCF-7, (**B**) MCF-7PR, (**C**) 4T1 and (**D**) 4T1PR cells were treated with different concentrations of PFL for 72 hours. Representative blots showing concentration dependent effect of PFL on HER2, β-catenin, TCF-1, TCF-4, p-GSK3β, c-Myc, cyclinD1, MMP-7, cleaved PARP and cleaved Caspase-3. Actin was used as a loading control. Each experiment was repeated at least three times.

To further elucidate the molecular mechanism of cytotoxicity of PFL, MCF-7, 4T1, MCF-7PR and 4T1PR cells were treated with various concentrations (0, 1.25, 2.5, 5 and 7.5 µM) of PFL for 72 hours. Our western blot results showed that expression of HER2, β-catenin and c-Myc were significantly reduced by PFL treatment in a concentration dependent manner in all the cell lines tested. We also observed notable inhibition of the downstream effector molecules of HER2/β-catenin pathway such as TCF-1, TCF-4, p-GSK3β and cyclin D1 in parent, as well as, in resistant cells (Fig. 5A–D). In addition, an increase in the levels of Cl-PARP and Cl-Caspase-3 was observed with PFL treatment indicating apoptosis in these cell lines. These results indicate that PFL was able to suppress all the oncogenic markers, which were found to be upregulated in paclitaxel resistant cells.

### PFL increases the sensitivity of 4T1PR and MCF-7PR cells towards paclitaxel

**Figure 6:**
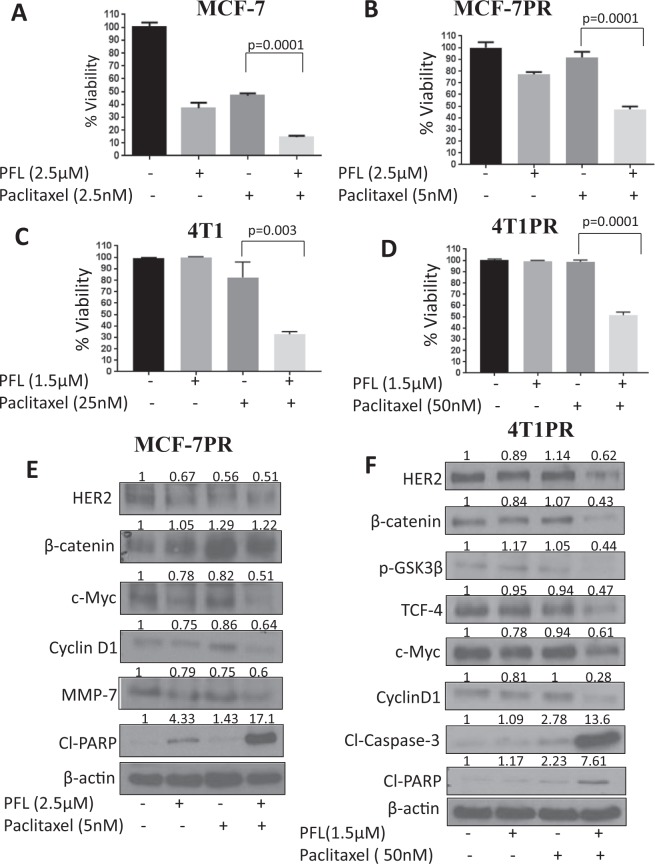
Pre-treatment with PFL sensitizes breast cancer cell lines to paclitaxel cytotoxicity. Percentage cell viability of breast cancer cells when pre-treated with PFL at indicated concentrations for 2 hours followed by paclitaxel treatment for 72 hours in (**A**) MCF-7 cells (**B**) MCF-7PR (**C**) 4T1 and (**D**) 4T1PR cells at indicated concentrations. Western blot showing a downregulation in resistant markers: HER2, β-catenin, c-Myc and an increase in Cl-Caspase-3 and Cl-PARP levels at a 2 hours pre-treatment combination of PFL and paclitaxel in (**E**) MCF-7PR cells and (**F**) 4T1PR cells analyzed 72 hours post paclitaxel exposure. Data shown as mean ± SD of at least three independent experiments, n = 3. All the statistical comparisons were made by Student’s *t* test for unpaired samples.

In order to determine whether PFL can potentiate the effects of paclitaxel and can overcome drug resistance, MCF-7 and MCF-7PR cells were pre-treated with 2.5 μM PFL for 2 hours followed by exposure to paclitaxel for 72 hours and cell viability was determined. Our results showed that the combination of PFL with paclitaxel significantly reduced the survival of cells when compared to paclitaxel alone (Fig. 6A,B). Combination of 1.5 μM PFL and 25 nM paclitaxel in 4T1 cells showed 65% (p = 0.003) reduction in cell viability when compared with control and PFL group (Fig. 6C). Similarly, in 4T1PR cells, combination of 1.5 μM PFL with 50 nM paclitaxel displayed 50% (p = 0.0001) decrease in cell survival than with either treatment alone (Fig. 6D). Combination index (CI) was calculated as described by us previously using Compusyn software^37,38^. We observed a very high degree of synergistic dug interaction (CI < 0.5) in these experiments. Concentrations as low as 0.5 μM PFL were able to sensitize the cells to paclitaxel-induced cytotoxicity (Supplementary Fig. 1). These findings were further confirmed by western blot analysis showing an increase in Cl-Caspase-3 and Cl-PARP levels in combination of 2.5 μM PFL with 5 nM paclitaxel in MCF-7PR (Fig. 6E) and with 1.5 μM PFL and 50 nM paclitaxel in 4T1PR cells (Fig. 6F). In addition, western blot analysis also showed considerable downregulation of resistant markers such as HER2, β-catenin, pGSK3β and c-Myc with treatment of paclitaxel and PFL combination in both the resistant cell lines (Fig. 6E,F). These observations complement cytotoxicity studies of drug combination and suggest a potential application of PFL as an adjuvant to repress resistance to paclitaxel and enhance its cytotoxic potential in breast cancer.

### Suppression of breast tumor growth by PFL and paclitaxel combination

**Figure 7:**
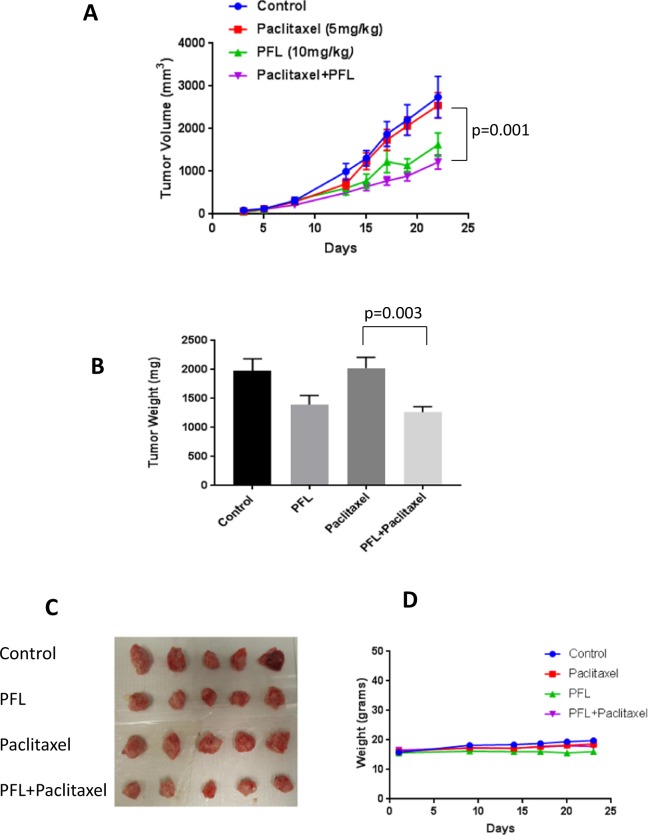
PFL and paclitaxel combination suppresses the growth of orthotropically implanted paclitaxel resistant breast tumor. (**A**) Analysis of tumor volume at various time points after orthotropically injecting approximately 0.07 × 10^6^ 4T1PR breast cancer cells in the left and right 3^rd^ mammary fat pads of 4–6 weeks old female Balb/c mice. Group I served as control and received the vehicle only. Group II received 10 mg/kg PFL by oral gavage everyday whereas group III received 5 mg/kg paclitaxel *(i.p*) every 3^rd^ day. Group IV received 10 mg/kg PFL every day and 5 mg/kg paclitaxel *(i.p)* every 3^rd^ day. Values were plotted as mean ± SEM (n = 10). (**B**) Analysis of tumor weight from different treatment groups. (**C**) Representative image showing tumors from different treatment groups. (**D**) Analysis of mice body weight from different treatment groups. Data shown as mean ± SEM. Statistical differences were calculated by Student’s *t* test.

To evaluate the efficacy of PFL and paclitaxel combination *in vivo*, highly aggressive 4T1PR murine breast tumor cells were implanted orthotropically in left and right 3^rd^ mammary fat pad of female Balb/c mice. Our results demonstrated that the group of mice receiving combination of PFL (10 mg/kg, every day) and paclitaxel (5 mg/kg, every 3^rd^ day) showed significant (p = 0.0012) tumor growth suppression (55%) when compared to paclitaxel, whereas group receiving PFL alone showed 40% of tumor reduction (Fig. 7A). Notably, Paclitaxel treatment alone showed almost no effect in suppressing tumor growth. Tumors were collected and weighed at the day of termination of experiment. Average weight of tumors in the combination group was about 40% (p = 0.003) less as compared with control group (Fig. 7B). In addition, there was no significant change in the mice weight throughout the study, suggesting no toxicity with drug combination. (Fig. 7D).

### PFL and paclitaxel combination suppresses HER2 and induces apoptosis *in vivo*

**Figure 8:**
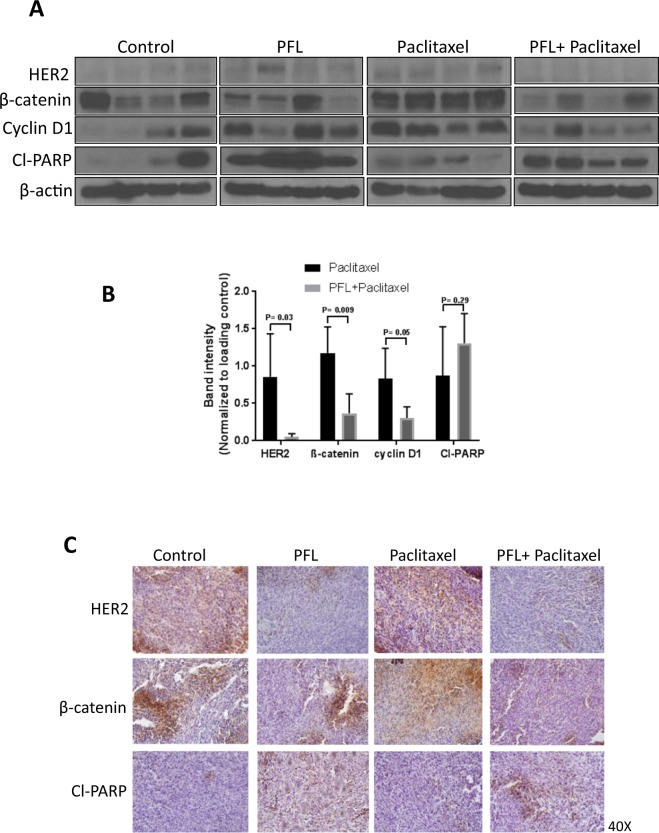
*S*uppression of HER2/β-catenin signaling by PFL in combination with paclitaxel *in vivo*. (**A**) Tumors were aseptically removed after terminating the experiments and lysed, homogenized and analyzed for HER2, β-catenin, cyclin D1 and cleaved PARP levels by Western blotting. Actin was used as a loading control, each lane of blots represents tumor from individual mouse. (**B**) Blots were quantified, normalized with actin, and represented as a bar graph. (**C**) IHC analysis of excised tumor sections for HER2, β-catenin and cleaved PARP.

In order to confirm the *in vitro* findings, tumors from mice from different groups were evaluated by western blot analysis. Tumors obtained from the combination group (PFL + paclitaxel) showed reduced expression of HER2, β-catenin and cyclin D1 as compared to tumors from mice treated with paclitaxel alone (Fig. 8A,B). Interestingly, we also observed that paclitaxel treatment alone in these mice resulted in the increased expression of HER2, β-catenin and cyclin D1 as seen by western blotting (Fig. 8A). Although, we did not see similar results with *in vitro* treatment of paclitaxel in 4T1PR cells (Fig. 6F). One explanation could be that long term exposure of paclitaxel is causing enhanced expression of these resistant markers *in vivo*. In agreement with our *in vitro* observations, our *in vivo* results also demonstrated increased levels of Cl-PARP in the tumors from mice treated with combination of PFL and paclitaxel as compared to paclitaxel treatment alone indicating apoptosis (Fig. 8A). These observations were further confirmed by immunohistochemical staining (IHC) of tumors from different groups for HER2, β-catenin and cleaved PARP. Our results demonstrated reduced expression of HER2 and β-catenin as well as increase in cleavage of PARP in combination group as compared to paclitaxel treatment alone (Fig. 8C).

## Discussion

Paclitaxel is a widely used chemotherapeutic agent for the treatment of various types of cancers: breast, ovary, lung and pancreas^39–42^. However, paclitaxel resistance is one of the primary obstacle leading to chemotherapy failure in breast cancer^43^. Investigating the molecular mechanisms responsible for chemotherapeutic resistance is highly desirable in identifying new drug targets. There are few mechanisms which have been reported to be involved in paclitaxel resistance; such as activation of PI3K/Akt, hedgehog/GSK3β, drug transporters and β tubulin mutation^9,44–46^.

In the present study, we developed paclitaxel-resistant MCF-7 and 4T1 cells to investigate the molecular mechanisms leading to acquired chemoresistance in breast cancer cells. Human epidermal growth receptor 2 (HER2) is an oncogene that plays an important role in the progression of aggressive breast cancer leading to poor disease prognosis^44–47^. The role of HER2 in taxane resistance is controversial and needs to be validated^17–19,21^. With that in mind, we explored the role of HER2 in inducing paclitaxel resistance. Hitherto, we observed that gradual exposure of breast cancer cells to paclitaxel over time leads to the upregulation of HER2 in low HER2 expressing MCF-7 and 4T1 cell lines. In addition, we observed increased sensitivity to paclitaxel in resistant cells after HER2 knockdown. On the other hand, paclitaxel sensitivity was reduced in cells with HER2 overexpression–indicating a crucial role of HER2 in inducing resistance towards paclitaxel in the forementioned cell lines.

Very few studies have shown the correlation of HER2 with β-catenin in HER2 positive breast cancer^22,23^. Herein, we observed a cross-talk between HER2 and β-catenin involved in inducing paclitaxel resistance. β-catenin is a proto-oncogene which imparts stem cell-like characteristics to cancer cells and is responsible for tumor progression and metastasis^48^. Literature suggests targeting Wnt/β-catenin signaling by inhibiting β-catenin or Mcl-1 enhances sensitivity towards taxanes in ovarian and prostate cancer^49,50^. In line with the existing results, we observed upregulation of β-catenin, c-Myc and other downstream molecules in breast cancer cell lines. Hence, we established that HER2/β-catenin mediates paclitaxel resistance in breast cancer and that suppression of HER2 and β-catenin signaling could overcome paclitaxel resistance.

Few studies including those from our laboratory have investigated the anti-cancer effects of PFL^35,36,51,52^. We have previously demonstrated that PFL suppresses metastatic breast tumor growth in the brain by inhibiting integrin signaling^35^. PFL is also known to induce ROS in breast cancer cells leading to apoptosis, concomitant with downregulation of Sp transcription factors^53^. Furthermore, PFL suppresses glioblastoma tumor growth by Akt mediated inhibition of Gli1^34^. However, none of the previous studies have investigated the effects of PFL in suppressing chemo-resistance, or modulation of HER2/β-catenin signaling in breast cancer. Our results showed that PFL was equally cytotoxic to paclitaxel-sensitive and paclitaxel-resistant cells. Treatment with PFL resulted in downregulation of chemo-resistant markers such as HER2, β-catenin, c-Myc and cyclin D1; with an increase in the pro-apoptotic markers such as Caspase-3 and PARP in MCF-7, MCF-7PR, 4T1 and 4T1PR cells. In addition, pretreatment with PFL restored the sensitivity of paclitaxel-resistant MCF-7 and 4T1 cells to paclitaxel as indicated by decreased cell viability in the combination treatment groups. Furthermore, treatment with PFL in combination with paclitaxel reduced tumor volume by more than 50% as compared to paclitaxel treatment alone. Paclitaxel treatment (5 mg/kg) alone does not seem to be effective at all in inhibiting tumor growth. However, PFL was able to suppress resistant tumor growth as a single agent as well as in combination with paclitaxel. Moreover, no change in mouse body weight was observed; indicating that the combination dose was effective and well tolerated. Dose of PFL used in the current study is 10 mg/kg, which when converted to human equivalent dose is 0.83 mg/kg. Therefore, human equivalent dose of PFL would be approximately 50 mg for a person weighing 60 Kg.

We demonstrate the novel role of β-catenin in conferring resistance to paclitaxel through HER2 overexpression in breast cancer. To the best of our knowledge, the present study provides a first evidence of the involvement of HER2/β-catenin signaling as a potential target in paclitaxel-resistant breast cancer, along with the observation that PFL was able to inhibit the expression of HER2, β-catenin and its downstream molecules in paclitaxel-sensitive and resistant cells. Our study also provides convincing results to establish a combination therapy of PFL with paclitaxel for the treatment of breast cancer–eventually leading to a reduction of the clinical dose of paclitaxel, consequently reducing the toxic side effects.

Taken together, PFL can reverse paclitaxel resistance by suppressing HER2/β-catenin signaling. These results not only provide the potential application of PFL as a novel therapeutic agent in sensitizing breast cancer cells to paclitaxel but also highlight the potential role of HER2/β-catenin signaling in the development of paclitaxel resistance in breast cancer.

## Materials and Methods

### Cell culture

Human breast cancer cell line MCF-7 and murine breast cancer cell line 4T1 were purchased from ATCC and were maintained in DMEM supplemented with 10% FBS and 5% PSN (Penicillin, Streptomycin, Neomycin) at 37 °C under a humidified atmosphere of 5% CO_2_. MCF-7HH cells were kindly provided by Dr. Huang Fei and maintained under similar conditions as MCF-7 cells. Resistance to paclitaxel in MCF-7 and 4T1 cells were developed in our laboratory and resistant cells were named as MCF-7PR and 4T1PR, respectively. The culture conditions for MCF-7PR and 4T1PR cells were same as those of the parent cell lines, with the exception of the addition of 8 nM and 50 nM paclitaxel respectively. All the cells used in this study are periodically authenticated by short tandem repeats (STR) analysis in our core facility.

### Development of paclitaxel resistant MCF-7 and 4T1 cells

MCF-7 and 4T1 cells were exposed to increasing concentrations of paclitaxel for several months. The treatment of MCF-7 cells started with 2.5 nM paclitaxel (half of the IC_50_) and was gradually increased to 300 nM over a period of 10 months. Similarly initial treatment of 4T1 cells was 4 nM paclitaxel (1/10^th^ of the IC_50_) that was increased to  300 nM over the period of 10 months. SRB assay was used to evaluate the cytotoxicity of paclitaxel in these cell lines and to confirm the resistance at different time points during the process.

### Cytotoxicity Studies

Cells were plated at a density of about 2000–4000 cells/well in 96 well plates and the treatment agents (PFL and/or paclitaxel) were added the following day. After desired duration of treatment (24, 48 and 72 h with PFL and 72 hours with paclitaxel), cells were fixed using 200 μl of ice cold 10% trichloroacetic acid followed by washing and staining with Sulforhodamine B (SRB) dye (0.4% SRB in 0.1% acetic acid). After incubation for 1 h at room temperature, plates were rinsed three times with 1% acetic acid and air-dried. Tris base solution (10 mM) was then added to the wells to solubilize the protein-bound dye and the optical density was measured using plate reader (BioTek Instruments, VT) as described by us previously^54^.

### Colony formation Assay

Approximately 500–600 cells/well were seeded in a 6-well plate. Next day, cells were treated with various concentrations of PFL. After 72 hours of PFL treatment, media was re-placed with fresh medium in all the wells and then cultured for another 5 days. At day 9, cells were fixed and stained with 0.5% crystal violet solution after washing with PBS. Finally, the colonies with >50 cells were counted under an image J-software.

### Immunofluorescence Analysis

Paclitaxel sensitive MCF-7 and paclitaxel resistant MCF-7 (MCF-7PR) cells were plated on a coverslip and allowed to attach overnight. The cells were then washed with ice-cold PBS twice and fixed with formalin and permeabilized using Triton-X100 solution. After blocking with 5% goat serum for 60 min, cells were incubated overnight with primary antibodies specific to HER2 or β-catenin at 4 °C, followed by incubation with Alexa Flour 594 secondary antibodies for 1 h by gentle rocking at room temperature. After washing the cells with PBS, nucleus was counterstained with DAPI and images were taken using fluorescence microscope (Olympus, Center Valley, PA) after mounting the coverslips on the slides.

### HER2 silencing

MCF-7PR cells were transfected with HER2 siRNA (Cell Signaling Technologies, Danvers, MA) using siPORT (Ambion Inc, Austin, TX) transfection reagent as per manufacturer’s protocol. Briefly, approximately 0.2 × 10^6^ cells/well were plated in 6 well plate and left overnight for attachment. Next day, cells were transfected with 100 nM HER2 siRNA or scrambled siRNA using siPORT reagent and 8 h post transfection, cells were treated with 20 nM of paclitaxel for additional 72 h. The cells were collected after treatment and processed for western blot analysis.

### Western Blot Analysis

The cells were harvested and rinsed twice with ice-cold PBS and whole cell lysates were prepared using 4% (w/v) CHAPS in urea-tris buffer. Cell lysates were kept on ice for 60 minutes, followed by sonication and centrifugation at 13,000 rpm for 20 minutes at 4 °C. The supernatant was collected and protein concentration was quantified using Bradford assay. Equal amount of protein (30–50 μg) were subjected to SDS-PAGE and resolved proteins were transferred to PVDF membrane. The membranes were probed for primary antibodies against HER2, β-catenin, pGSK3β, TCF-1, TCF-4, Cyclin D1, c-Myc, Cl-PARP, Cl-Caspase-3, β-actin. All primary antibodies were purchased from Cell Signaling Technologies (Danvers, MA) except β-actin which was obtained from Sigma Aldrich, (St Louis, MO). The membranes were developed as described by us previously^37,54^.

### Equipment and settings

Western blots were developed using X-ray film based on the principle of chemiluminescence (ECL) substrates for horseradish peroxidase (HRP). Films were scanned and blots were quantified using UN-SCAN-IT gel 7.1 software. No post-processing was done (eg. Photoshop, brightness contrast changes etc.). Different markers of the same figure were either obtained from the same experiment or that gels/blots were processed in parallel. Full length blots including different exposure times are provided in Supplementary Figs 2–6. All the animal experiment protocols were approved by Institutional Animal Care and Use Committee (IACUC), Texas Tech University Health Sciences Center (Amarillo, Texas).

### Orthotropic breast tumor model

Female Balb/c mice (4–6 weeks old) purchased from Envigo (Indianapolis, IN, USA) were used for this study. Exponentially growing 4T1 paclitaxel resistant cells were harvested, washed twice with PBS and re-suspended in 1:1 PBS/matrigel at a density of 0.7 × 10^6^ cells/ml. Further, 0.07 × 10^6^ cells were implanted in the left and right 3^rd^ mammary fat pads of each mouse. Tumor volumes were measured three times a week using Vernier Calipers and animal weights were taken twice a week. Tumor volume was calculated using the formula (length × (width)^2^/2). When tumor volume reached 70–100 mm^3^ (day 4), mice were randomly divided into four groups with 5 mice per group. Group I served as control and received vehicle only. Group II was administered with 10 mg/kg PFL by oral gavage daily, whereas group III received 5 mg/kg paclitaxel *(i.p)* every 3^rd^ day. Group IV was administered with 10 mg/kg PFL daily as well as 5 mg/kg paclitaxel (*i.p*) every 3^rd^ day respectively. Mice were euthanized on day 23^rd^ with CO_2_ overdose and death was confirmed by cervical dislocation in accordance with IACUC guidelines. The tumors were removed aseptically from each mouse, weighed and snap frozen in liquid-nitrogen for western blot analysis or fixed in formalin for IHC analysis. Experiments were conducted in strict compliance with the regulations of IACUC, Texas Tech University Health Sciences Center.

### Immunostaining of tumor sections

The IHC was done as previously described by us^55^. Tumors collected from *in vivo* study were dehydrated, embedded in paraffin and sectioned into 5–10 micron sections using microtome. The sections were gently placed on positively charged slides, deparaffinized and rehydrated using xylene, ethanol and double-distilled water. The sections were further boiled in 10 mM sodium citrate buffer (pH 6.0) for antigen unmasking and incubated in 3% hydrogen peroxide solution. After blocking with 5% goat serum, sections were incubated overnight with primary antibodies for HER2, β-catenin and cleaved PARP. Next day, using Ultravision ONE HRP polymer kit (Thermofisher scientific, Fremont, CA), the slides were stained as per the manufacturer’s instructions. Further, these sections were counterstained with Mayer’s hematoxylin, dehydrated and mounted using Permount and then imaged using Olympus microscope (Olympus America Inc, Center Valley, PA).

### Statistical Analysis

Experiments were repeated at least thrice and represented as mean ± SD or SEM. Student’s *t* test was used to compare the statistical significance between two groups. Statistical significance was calculated using the using Prism 7.0 (GraphPad software Inc., San Diego, CA, USA). A *p* value of less than 0.05 was considered statistically significant (*).

## Supplementary information

Supplementary Figure 1
