# Supplementary material for: Penfluridol overcomes paclitaxel resistance in metastatic breast cancer
Source: Sci Rep. 2019 Mar 25;9:5066. doi: 10.1038/s41598-019-41632-0 (PMC6434141; doi:10.1038/s41598-019-41632-0)
Supplement: Supplementary file 1 — Supplementary Figure 1 [file 41598_2019_41632_MOESM1_ESM.pdf]

Penfluridol overcomes paclitaxel resistance in metastatic breast cancer by targeting inhibiting HER2/β-Catenin Signaling

Nehal Gupta[1], Parul Gupta[1,2] and Sanjay K. Srivastava[*1, 2]

[1]Department of Biomedical Sciences, Texas Tech University Health Sciences Center, Amarillo, TX 79106, USA;

[2]Department of Immunotherapeutics and Biotechnology, Texas Tech University, Abilene, TX, USA

[*]**Corresponding author:** Sanjay K. Srivastava, Ph.D., Department of Immunotherapeutics and Biotechnology, Texas Tech University Health Sciences Center, Suite 1305, 1718 Pine Street, Abilene, Texas 79601. Phone: 325-696-0464; Fax: 806-356-4770; E-mail: sanjay.srivastava@ttuhsc.edu

Supplementary Figure: 1

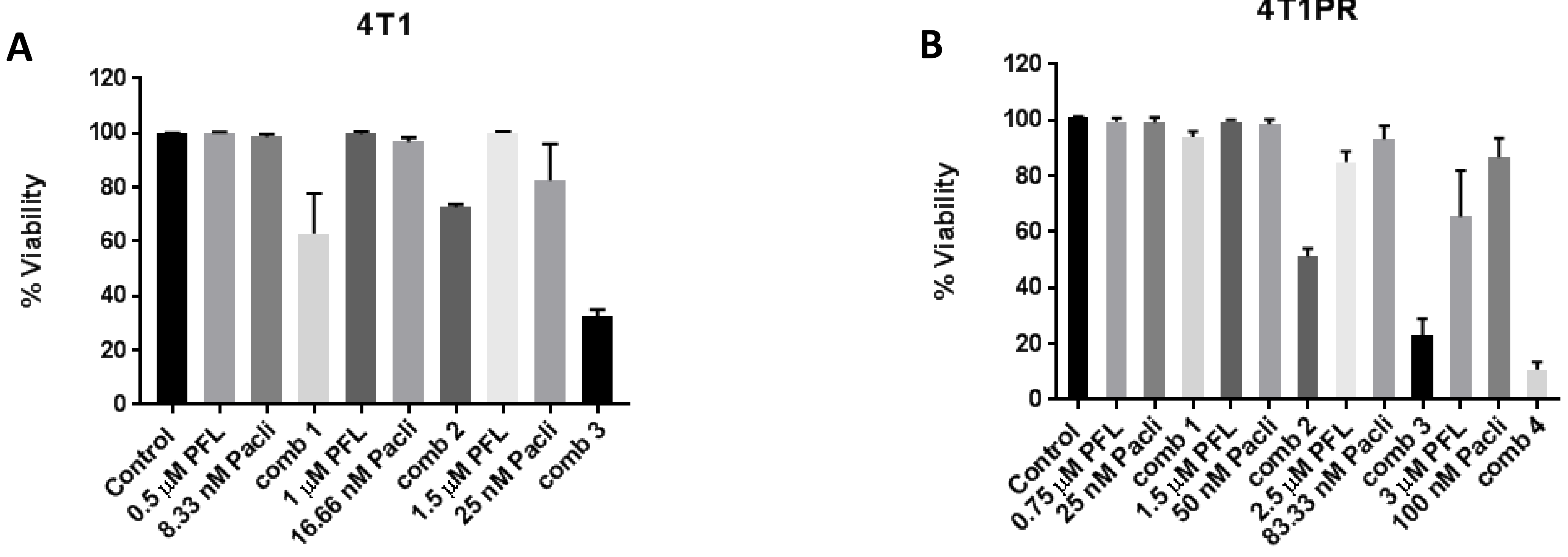

4T1		4T1PR	
CI	DRI	CI	DRI
0.0006	>100	0.321	>100

Supplementary Figure:1 Pretreatment with **PFL** followed by Paclitaxel (Pacli) exposure for 72 hours in (A) 4T1 cells and (B) 4T1PR cells. Insert depicts the parameters for quantifying the degree of synergism. Data shown as mean ± SD; n=4.

CI: Combination index -Value less than one shows synergism

DRI: Dose reduction index- reduction in dose of paclitaxel to kill breast cancer cells when used in combination with paclitaxel

Supplementary Figure: 2

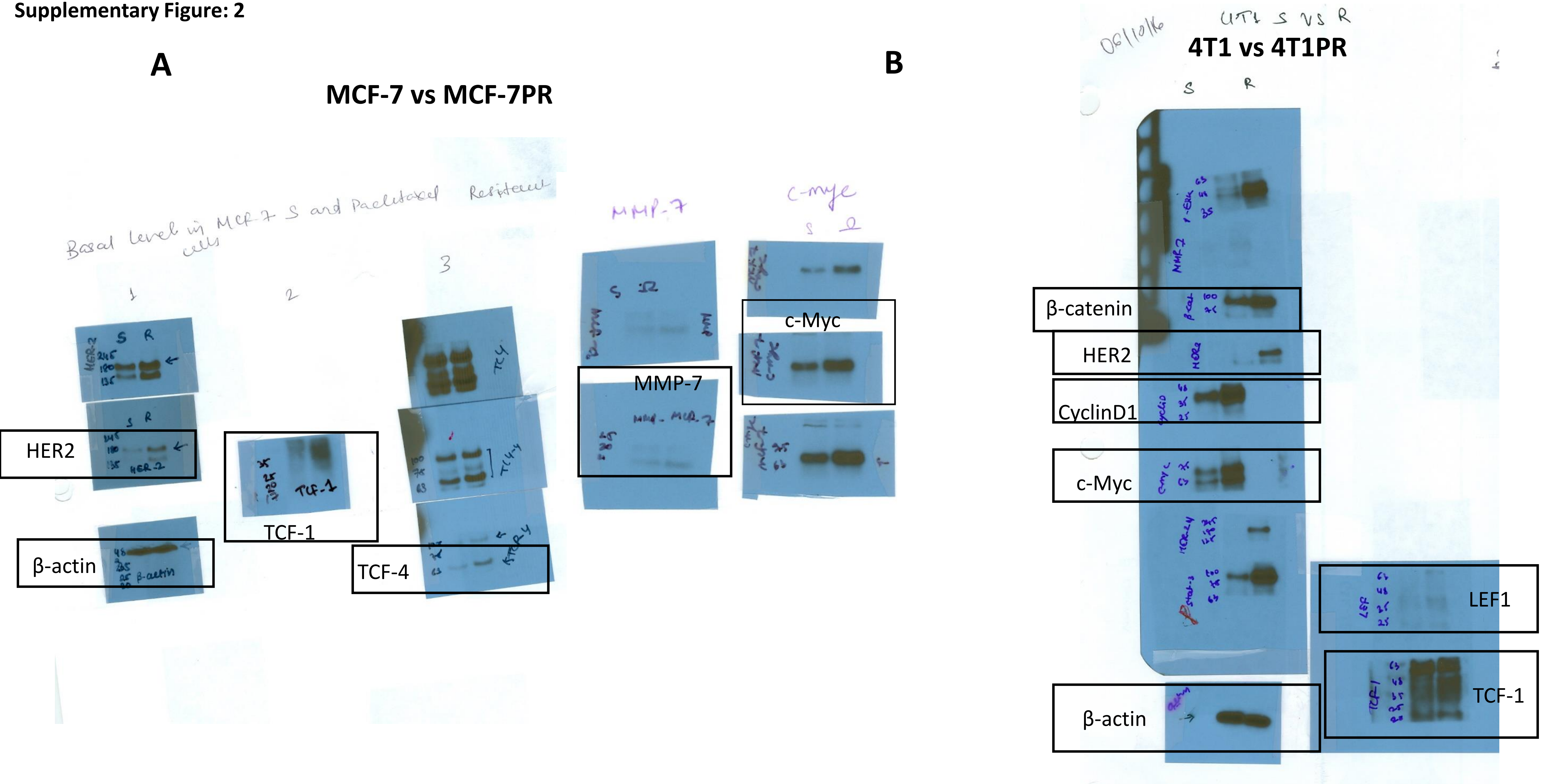

Supplementary figure 2: Full length western blot images. A) Full length western blots of in MCF-7 and MCF-7PR cells. Cropped images and description shown in Figure 1C. B) Full length western blots of in 4T1 and 4T1PR cells. Cropped images and description shown in Figure 1D

Supplementary Figure: 3

MCF-7PR

A

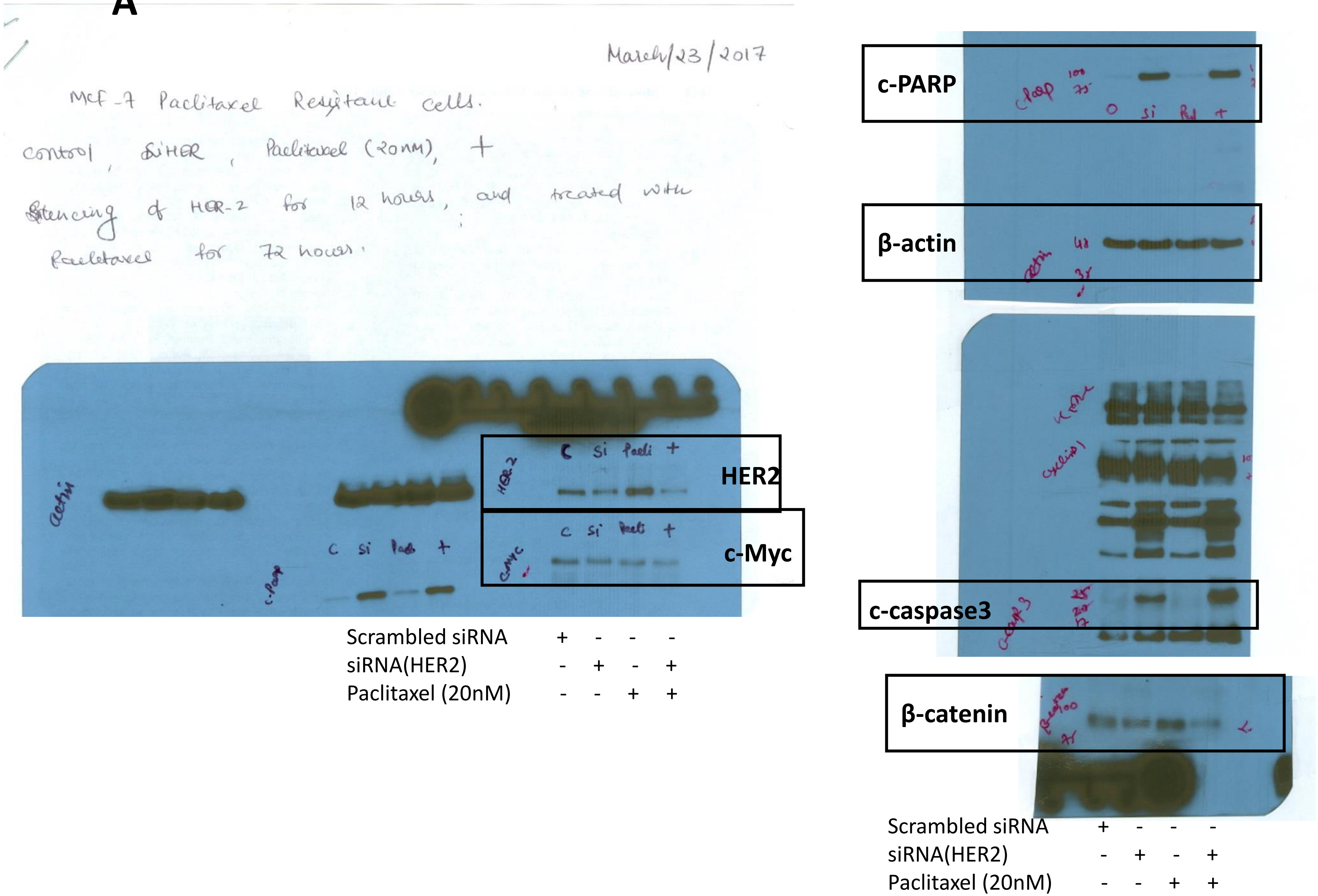

Supplementary figure 3: Full length western blot images for figure 2C. MCF-7PR cells were transfected with either HER2 siRNA or scrambled siRNA for 8 hours and then treated with paclitaxel (20nM) for additional 72 hours.

Supplementary Figure: 4

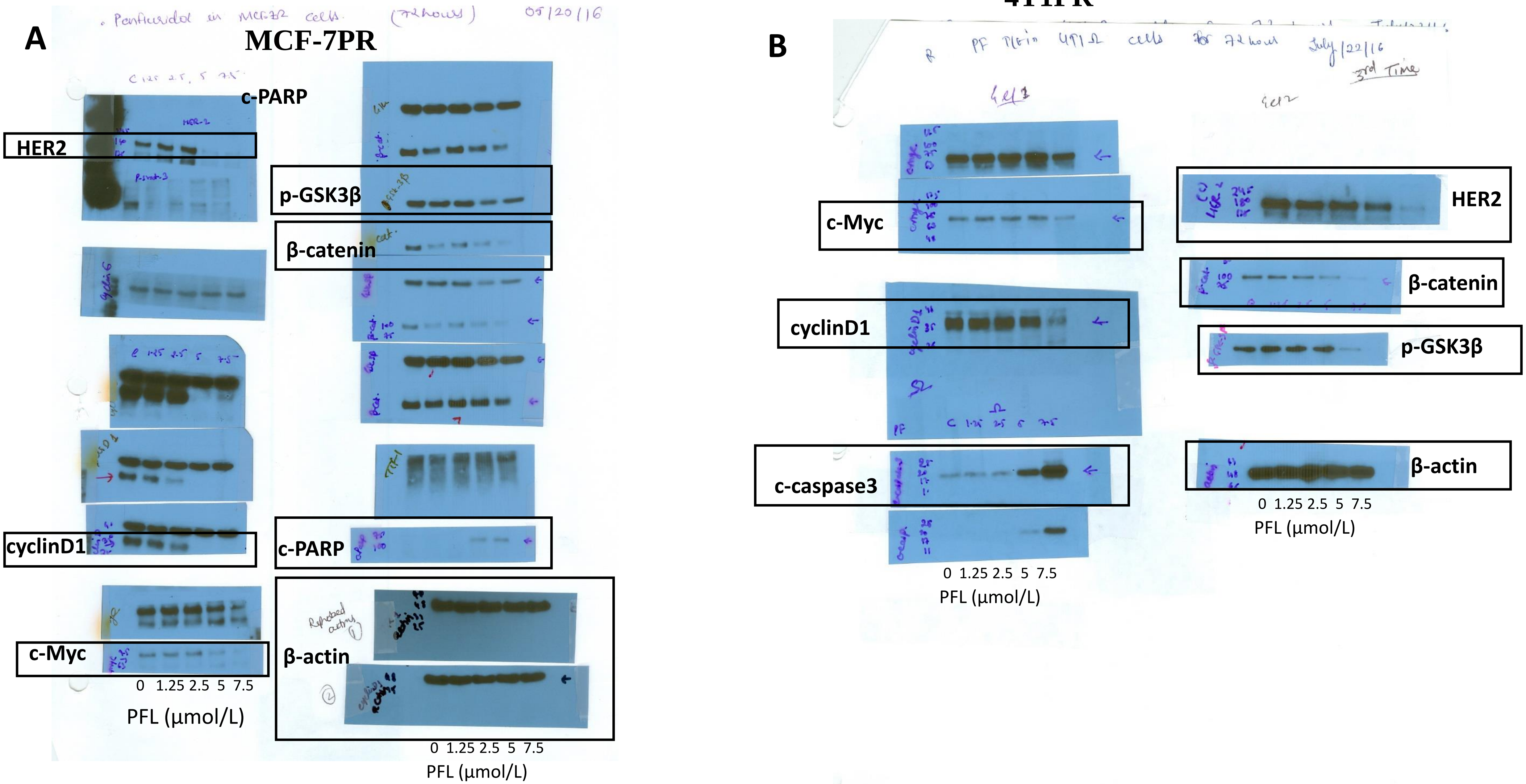

Supplementary figure 4: Penfluridol treatment at indicated concentrations for 72 hours in A) MCF-PR and B) 4T1PR cells. Cropped images and description shown in Figure 5B and 5D.

Supplementary Figure: 5

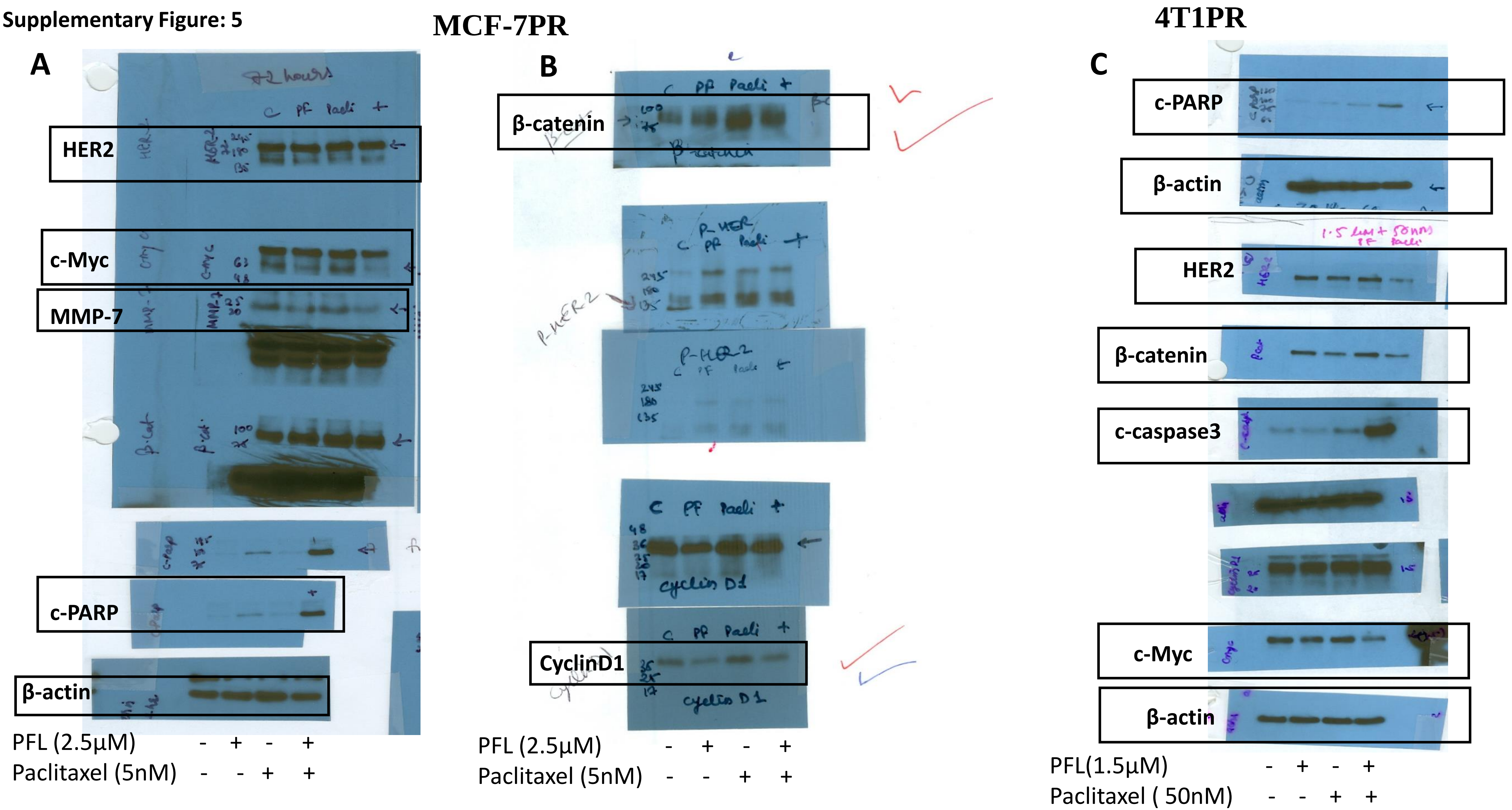

Supplementary figure 5: Full length western blot images. A&B) Full length western blots of in MCF-7PR cells. Cells were pretreated with 2.5µM PFL for 2 hrs and further treated with 5 nM paclitaxel for 72 hrs. Cropped images and description shown in Figure 6E. C) Full length western blots of 4T1PR cells treated with 1.5µM PFL and 50nM of paclitaxel . Cropped images and description shown in Figure 6F.

Supplementary Figure: 6

4T1PR tumor lysates

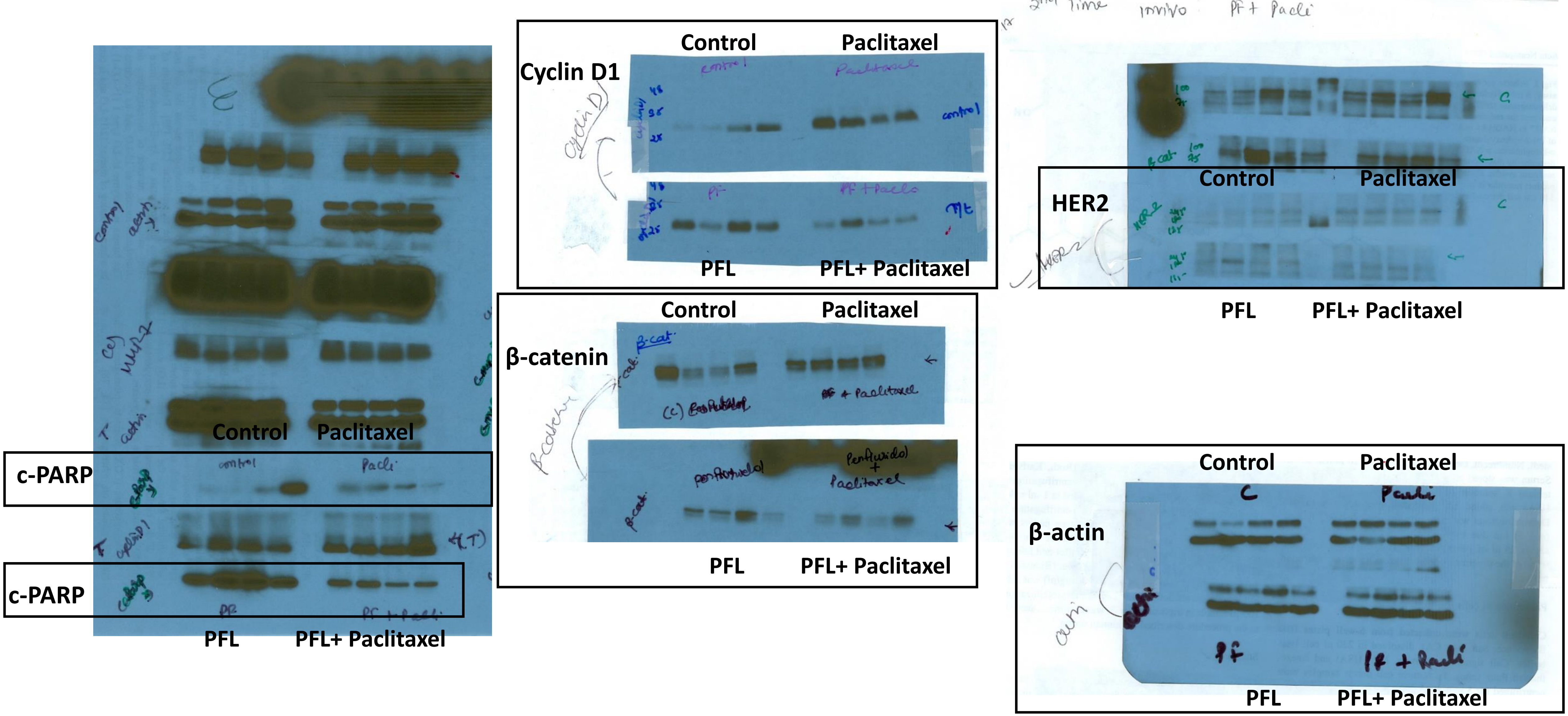

Supplementary figure 6: Full length western blot images for *in vivo* tumor lysates. Cropped images and description shown in Figure 8A.
